# Hybrid Hydrogel Composed of Polymeric Nanocapsules Co-Loading Lidocaine and Prilocaine for Topical Intraoral Anesthesia

**DOI:** 10.1038/s41598-018-36382-4

**Published:** 2018-12-19

**Authors:** Bruno Vilela Muniz, Diego Baratelli, Stephany Di Carla, Luciano Serpe, Camila Batista da Silva, Viviane Aparecida Guilherme, Lígia Nunes de Morais Ribeiro, Cintia Maria Saia Cereda, Eneida de Paula, Maria Cristina Volpato, Francisco Carlos Groppo, Leonardo Fernandes Fraceto, Michelle Franz-Montan

**Affiliations:** 1 Department of Physiological Sciences, Piracicaba Dental School, University of Campinas – UNICAMP, Piracicaba, São Paulo Brazil; 2 São Paulo State University – UNESP, Institute of Science and Technology of Sorocaba, Department of Environmental Engineering, Sorocaba, São Paulo Brazil; 3 Department of Biochemistry and Tissue Biology, Institute of Biology, University of Campinas – UNICAMP, Campinas, São Paulo Brazil

## Abstract

This study reports the development of nanostructured hydrogels for the sustained release of the eutectic mixture of lidocaine and prilocaine (both at 2.5%) for intraoral topical use. The local anesthetics, free or encapsulated in poly(ε-caprolactone) nanocapsules, were incorporated into CARBOPOL hydrogel. The nanoparticle suspensions were characterized *in vitro* in terms of particle size, polydispersity, and surface charge, using dynamic light scattering measurements. The nanoparticle concentrations were determined by nanoparticle tracking analysis. Evaluation was made of physicochemical stability, structural features, encapsulation efficiency, and *in vitro* release kinetics. The CARBOPOL hydrogels were submitted to rheological, accelerated stability, and *in vitro* release tests, as well as determination of mechanical and mucoadhesive properties, *in vitro* cytotoxicity towards FGH and HaCaT cells, and *in vitro* permeation across buccal and palatal mucosa. Anesthetic efficacy was evaluated using Wistar rats. Nanocapsules were successfully developed that presented desirable physicochemical properties and a sustained release profile. The hydrogel formulations were stable for up to 6 months under critical conditions and exhibited non-Newtonian pseudoplastic flows, satisfactory mucoadhesive strength, non-cytotoxicity, and slow permeation across oral mucosa. *In vivo* assays revealed higher anesthetic efficacy in tail-flick tests, compared to a commercially available product. In conclusion, the proposed hydrogel has potential for provision of effective and longer-lasting superficial anesthesia at oral mucosa during medical and dental procedures. These results open perspectives for future clinical trials.

## Introduction

Topical anesthetics are applied superficially to reduce or control pain in medical and dental procedures such as local anesthetic injection, placement of orthodontic bands, simple extraction of primary teeth, rubber-dam clamp placement, biopsies, abscess incision, endotracheal intubation, and endoscopy^1^. Nevertheless, variables such as the class and concentration of the anesthetic agent, pH, additives, contact time at the mucosa, duration of action, and site of application influence the success of superficial anesthesia^2^.

Lidocaine (LDC) and prilocaine (PLC) are amine-amide local anesthetics (LAs) widely used in biomedical procedures worldwide^1^. When these LAs are combined, they form an eutectic mixture that is commercially available as EMLA, a topical formulation originally designed for dermal use, with proven effectiveness inside the oral cavity^3^. However, the formulation also presents organoleptic characteristics such as a bitter taste (pH = 9.0) and a burning sensation during application, which can hinder its acceptance by patients^2^. The advantages and disadvantages of EMLA led to the development of new formulations containing the eutectic mixture of LDC and PLC in drug delivery systems such as mucoadhesive films and nanostructured lipid carriers, aiming at topical oral application^4,5^.

Several approaches have been adopted for optimization of the effective loading of LAs into polymeric nanoparticles. The encapsulation of benzocaine, lidocaine, and articaine in polymeric nanoparticles composed of poly(lactide-co-glycolide), poly-L-lactide, and poly-ε-caprolactone resulted in long-term stability, sustained release, and increased anesthetic efficacy *in vivo*^6–11^.

Polymeric nanocapsules consist of an oily core and an ultrathin polymeric wall, providing particles smaller than 1 μm^12^. Poly-ε-caprolactone (PCL) is among the biodegradable polymers most widely employed for the preparation of nanocapsules, due to its desirable properties for incorporation in semisolid drug delivery systems, such as hydrophobicity and biocompatibility^13^.

Hydrogels are three-dimensional polymer networks cross-linked by physical or chemical agents, which can absorb large amounts of biological fluids. Their useful properties include biocompatibility, flexibility, and suitable rheological behavior, enabling their use in a wide range of applications including wound healing and topical delivery of active molecules at the skin and mucosa^14,15^. CARBOPOL is an acrylic acid copolymer employed as a matrix in several semisolid formulations, with interesting properties such as mucoadhesion, which promotes adherence to the mucus layer, leading to prolonged residence times of incorporated drugs^15,16^. CARBOPOL formulations containing benzocaine^17^, lidocaine^18^, or ropivacaine^19–21^ encapsulated in liposomes were shown to be effective in promoting topical anesthesia in the human oral mucosa.

The objective of the present study was to determine whether a hybrid system based on poly(ε-caprolactone) nanocapsules in CARBOPOL hydrogel could increase the biocompatibility, permeation capacity, and anesthetic efficacy of 5% lidocaine-prilocaine, aiming at topical intraoral anesthesia.

## Results and Discussion

### Characterization of poly(ε-caprolactone) nanocapsules

The encapsulation of different LAs using other polymeric nanocapsules has been described previously^7,22–24^, with the aim of prolonging analgesia and minimizing side effects^25^. Particle size and polydispersity index (PDI) are critical parameters of nanostructured systems that influence drug encapsulation efficiency, formulation stability, and release behavior^26^. The surface charge of nanoparticles (zeta potential, ZP) can be used as a predictive index of particle stability, as well as to elucidate the location of the active molecules in the nanocapsule^27^. The nanoparticle concentration is another parameter that affects the stability and biological activity of drug delivery systems^28^.

The results obtained for particle size, PDI, ZP, nanoparticle concentration, and pH, according to time, for nanocapsules with and without LAs, are provided in Fig. S1 (Supplementary Material). For the freshly prepared samples (Day 0), the analyzed parameters of the suspensions containing LDC + PLC were similar to those reported elsewhere for poly(ε-caprolactone) nanocapsules containing LAs^11^.

The nanocarrier showed high encapsulation capacities for both LDC (83 ± 3%) and PLC (72 ± 3%), indicating its superior performance, compared to polymeric nanospheres that usually present lower encapsulation efficiencies due to the absence of the oily inner core^29^. The higher EE% for LDC than for PLC could be explained by the higher octanol/water partition coefficient of LDC (P = 304), compared to PLC (P = 129), for the molecules in their neutral forms^30^. Hence, PLC would be more likely to interact with the aqueous phase and the polymer matrix of the nanocapsules, while LDC would exhibit better interaction with the oily nucleus^6^, due to its greater hydrophobicity.

The stability of the formulation during 180 days of storage at room temperature (25 °C) is also shown in Fig. S1. Changes in the mean diameter of the nanocapsules were detected after 90 days of storage (*p* < 0.05). The PDI values confirmed that the monodisperse size distribution was maintained until the end of the experiment, as required for this type of formulation. The stability results confirmed evidence in the literature showing that biodegradation of PCL is slow, compared to other polymers, and usually starts after 1 year^13^.

Another factor indicating the stability of the system was the high ZP modulus, reflecting repulsion among the nanocapsules in suspension^26^. The nanoparticles containing LDC and PLC presented satisfactory ZP values throughout the storage period, with continued inter-particle repulsion that could be attributed to the presence of PVA, which could form a barrier around the nanocapsules, resulting in steric stabilization^9^.

Nanoparticle tracking analysis (NTA) is a technique for monitoring the physicochemical stability of nanoparticulate systems that can provide unique information concerning the particle concentration^28^. In the present case, there were no significant changes in nanoparticle concentration (*p* > 0.05) throughout the study period, corroborating the other stability parameters determined by DLS, hence confirming the stability of the system.

**Figure 1:**
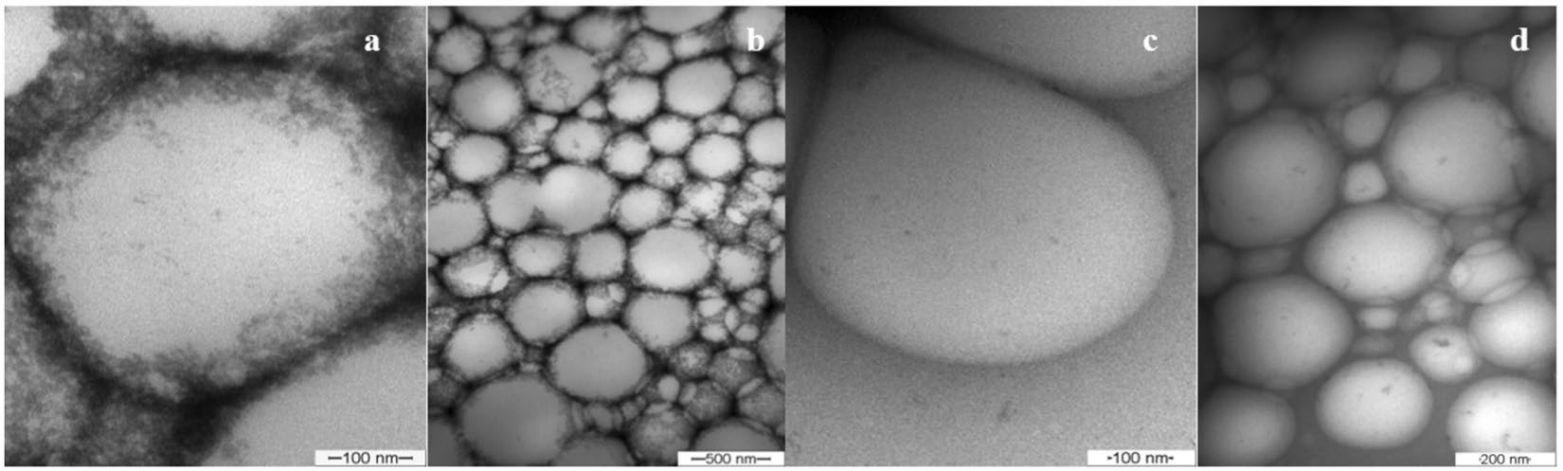
Transmission electron micrographs of PCL with (**a**,**b**) and without (**c**,**d**) LDC + PLC, at two different magnifications (10,000x and 60,000x).

The pH decreased over time (*p* < 0.01), as reported previously^9^, indicating degradation of the polymers and formation of acidic products by hydrolysis of the polymer chains, suggesting a possible lack of stability and breakdown of the nanocapsules. However, considering the observed stability of the particle size distribution, the polymer hydrolysis did not affect the structural properties of the nanoparticles. The morphology of the nanocapsules was confirmed by transmission electron microscopy (Fig. 1).

The micrographs revealed some differences between the nanocapsules with and without LDC + PLC. The spherical core-shell structure for the nanocapsules with the LAs (PCL/LDC + PLC) (Fig. 1a,b) suggested that the LDC and PLC bases were incorporated into the hydrophobic core, as previously reported for lidocaine-loaded poly(ε-caprolactone)-poly(ethylene glycol) nanoparticles^31^.

**Figure 2:**
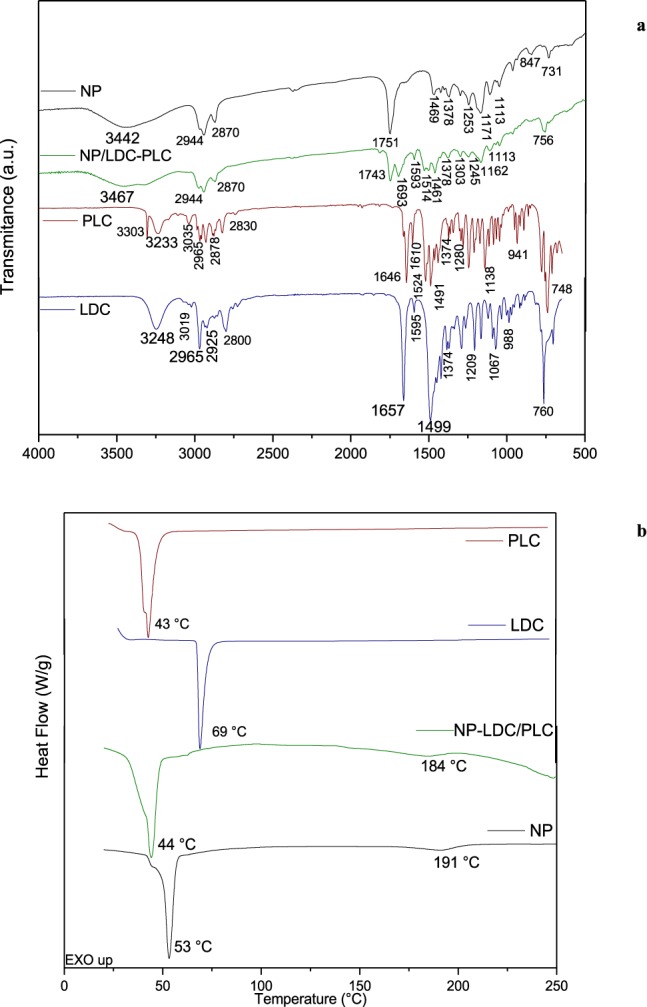
FTIR-ATR spectra (**a**) and DSC analysis (**b**) of the poly(ε-caprolactone) nanocapsules (NP and NP/LDC-PLC), lidocaine (LDC), and prilocaine (PLC).

The ATR-FTIR technique was used to investigate the interactions among the nanocapsules and the LAs. The FTIR-ATR spectra (Fig. 2a) of the poly(ε-caprolactone) nanocapsules (denoted NP) and the LAs showed typical bands of the molecules^5^. In the spectrum of the NP formulation, a band at 1580-1450 cm^−1^ could be attributed to C = C vibrations, while a band at 1735-1650 cm^−1^ corresponded to C = O and a band at 3000-2800 cm^−1^ was related to C-H of saturated carbons^22,23^. The spectroscopic profile of the polymeric nanoparticles remained similar after encapsulation of PLC + LDC. The bands in the region 2950–3305 cm^−1^ for the pure LAs completely disappeared in the spectrum of the NP/LDC + PLC formulation, indicating dissolution of the compounds in the nanocapsule core and the maintenance of particle integrity.

DSC is a thermoanalytical technique that can provide information concerning polymorphic changes in materials^32^. The calorimetry results (Fig. 2b) revealed the endothermic peaks corresponding to the melting points of the substances at 43 °C (PLC), 69 °C (LDC), and 53 °C (PCL)^5,33^. The PCL/LDC-PLC formulation showed a lower melting point (44 °C), compared to PCL, confirming the interaction among the components.

Figure S2 (Supplementary Material) shows the *in vitro* release of LDC and PLC from the PCL/LDC + PLC formulation, compared to the LAs free in solution. A burst release effect was observed for the non-encapsulated LAs, with 50% release of the drugs within the first 30 min. Complete release of the free LAs occurred within 4 h, while the release of the encapsulated drugs did not reach 100%, even after 18 h. The faster release of PLC was in agreement with its lower encapsulation efficiency, compared to LDC.

As shown in Table S1 (Supplementary Material), the mathematical model that provided the best description of LDC and PLC release from the nanocapsules was the semi-empirical Korsmeyer-Peppas model, which is commonly applied to nanostructured systems. This mathematical model has been used previously to describe the release of LDC and PLC loaded in polymeric nanospheres^9^. Based on the values of the release exponent (n), the release of the LAs from the nanocapsules could be characterized as being due to non-Fickian anomalous transport (0.45 < n < 0.89). Hence, there was more than one diffusion mechanism involved, with rapid diffusion of non-encapsulated LAs and slow diffusion of encapsulated LAs, with the latter requiring the compounds to cross the physical polymer barrier in order to be released^34^.

### Characterization of the hydrogels

#### Rheological studies

**Figure 3:**
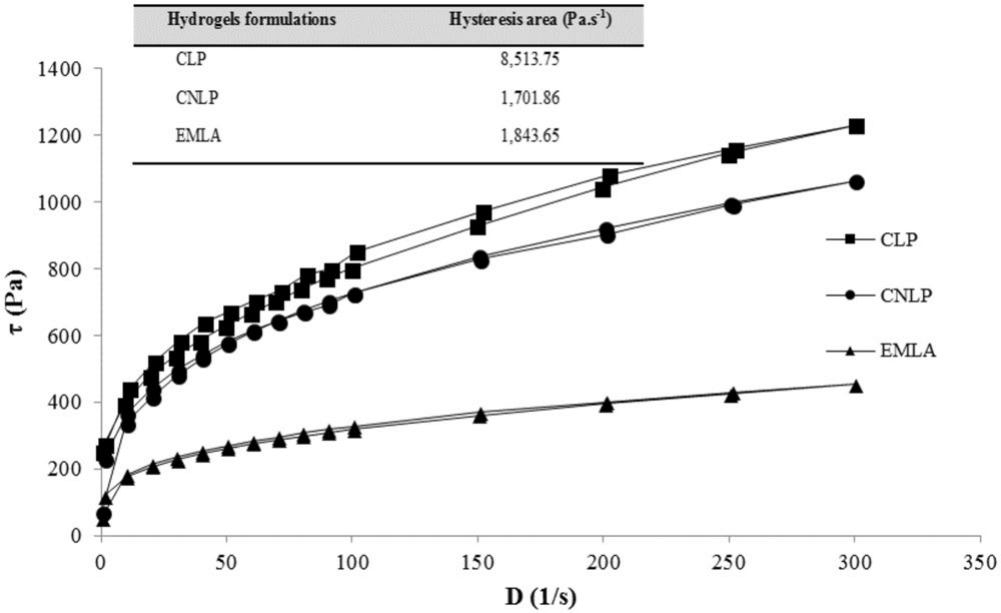
Rheological profiles showing the shear stress as a function of shear rate for the CARBOPOL hydrogels containing LDC and PLC (5%), free or associated with the poly(ε-caprolactone) nanocapsules (CLP and CNLP, respectively), in comparison with the commercial formulation composed of the eutectic mixture of LDC and PLC (EMLA). The inset shows the hysteresis areas. For each formulation, the upper and lower curves correspond to ascending and descending measurements, respectively. The SD values were below 5%.

Rheological analysis can be used to assist in elucidating the potential of innovative pharmaceutical formulations^35^. Figure 3 shows the rheological behaviors (flow curve profiles) of CARBOPOL hydrogels containing LDC and PLC (5%), free or associated with the poly(ε-caprolactone) nanocapsules (CLP and CNLP, respectively), in comparison to EMLA.

For all formulations, the rheograms reflected non-Newtonian pseudoplastic flows. The viscosity depends on the shear rate^36^. In other words, when the shear rate increases with increasing shear stress^35^, typical properties of semi-solid formulations are observed^37^.

Thixotropy is a rheological indicator of viscosity. This parameter can be calculated by measuring the area contained in the hysteresis loop of the rheological curves^35^, as shown in Fig. 3. A larger hysteresis area was found for the non-encapsulated LDC-PLC CARBOPOL hydrogel, suggesting delayed recovery of its structure^38^. An intermediate hysteresis area value is desirable in order to improve the shelf-life and provide good spreadability of semisolid formulations, as observed for the CNLP hydrogel and EMLA. Knowledge of thixotropic properties can help in increasing the retention times of topically applied formulations, leading to better therapeutic efficacy^35^.

#### Mechanical and mucoadhesive properties

The mechanical properties obtained by texture profile analysis (TPA) for all the hydrogels are shown in Table 1.

**Table 1:** Mean (±SD) values obtained for the mechanical properties and mucoadhesive strengths of CARBOPOL hydrogels containing LDC and PLC (5%), free or encapsulated in poly(ε-caprolactone) nanocapsules (CLP and CNLP, respectively), in comparison with EMLA.

Mechanical properties					Mucoadhesive strength
Formulation	Hard. (N)	Comp. (N.mm)	Cohes.	Adhes. (N.mm)	Detachment force (N)
CNLP	0.383 ± 0.028^a^	1.390 ± 0.168^a^	0.694 ± 0.016^a^	0.165 ± 0.021^a^	0.024 ± 0.005^a,b^
CLP	0.186 ± 0.011^b^	0.727 ± 0.040^b^	0.747 ± 0.027^b^	0.123 ± 0.006^b^	0.022 ± 0.004^a^
EMLA	0.133 ± 0.007^c^	0.500 ± 0.030^c^	0.852 ± 0.016^c^	0.173 ± 0.006^a^	0.032 ± 0.004^b^

Hard.: hardness; Comp.: compressibility; Cohes.: cohesiveness; Adhes.: adhesiveness. Different letters indicate significant differences among the hydrogels for each parameter evaluated (ANOVA/Tukey-Kramer test, *p* < 0.05). Each parameter was analyzed separately (n = 5).

An ideal semisolid formulation for use on the oral mucosa should have properties that enable easy application and spreading, determined by hardness and compressibility, respectively. Its permanence at the desired site without disintegrating can be predicted by higher values of adhesiveness and cohesiveness^39,40^.

The TPA results indicated that the CNLP hydrogel possessed good mechanical properties^41,42^. The presence of the poly(ε-caprolactone) nanocapsules increased hardness (*p* < 0.05) and compressibility (*p* < 0.05), with the values obtained being within the range reported to be ideal for topical oral application^43^. The cohesiveness and adhesiveness values were also satisfactory and were comparable to those reported for other semisolid formulations designed for topical application to the oral mucosa^40,44^.

The mucoadhesive strength, evaluated in terms of the detachment force, represents the force required to detach the hydrogel from the mucosal surface. The detachment force obtained for CNLP was similar to that for the commercial formulation (*p* > 0.05) and higher than for CLP (*p* < 0.05) (Table 1). These results indicated that the presence of the nanocapsules did not affect the mucoadhesive properties of CARBOPOL, in agreement with the findings of Frank *et al*.^45^, who also observed that the presence of poly(ε-caprolactone) nanocapsules did not alter the mucoadhesive capacities of carboxymethylcellulose and chitosan gels.

#### Accelerated stability study

Accelerated stability testing was used to observe possible physicochemical changes (weight loss, LA dosage, and pH) that could occur during storage of the hydrogels, as a result of degradation of components of the formulation. The study was performed at constant controlled temperature (40 ± 2 °C) and humidity (75 ± 5% RH), for up to 6 months, according to the ICH procedure^46^. As can be seen in Table S2 (Supplementary Material), storage did not affect the physicochemical properties of the CNLP formulation (*p* > 0.05), indicative of good stability.

#### *In vitro* release kinetics and mathematical modeling

The profiles of LDC and PLC release from the hydrogels are shown in Figs S3(a,b), respectively. The release kinetics data, as evaluated by the determination coefficient values of the applied mathematical models, are shown in Table S3 (Supplementary Material).

The Weibull mathematical model provided the best description of the release mechanism (R^2^ ≥ 0.946). This model consists of an empirical equation that is often used to describe the process of drug delivery using spherical media^47^. Based on the “b” value, the release of LDC in the absence of nanocapsules (CLP, b = 0.95) was according to a non-Fickian process (0.75 < b < 1) involving a combination of more than one release mechanism. This indicated that the local anesthetic was associated with the CARBOPOL structure and depended on relaxation of the structure for its release and subsequent diffusion to the external environment. In the case of PLC (CLP, b = 0.13), the compound showed a disordered distribution in the spaces between the three-dimensional structures of the gel (b < 0.35). In the presence of the nanocapsules (CNLP), the release of LDC (b = 0.72) changed to Fickian release (0.69 < b < 0.75), indicating that it occurred by diffusion between the gel layers. However, in this formulation, the release of PLC (b = 0.93) changed to non-Fickian transport (0.75 < b < 1), indicating the existence of two release mechanisms, with swelling and relaxation of the nanocapsule polymers, followed by subsequent diffusion from the hydrogel to the external medium^48^.

#### *In vitro* permeation studies

The parameter values for permeation of LDC and PLC from the different hydrogel formulations across oral epithelia (palatal = keratinized model; buccal = non-keratinized model) are shown in Table 2.

**Table 2:** Mean (±SD) values of the steady-state flux (J_ss_) and lag time for permeation of lidocaine (LDC) and prilocaine (PLC), free or associated with poly(ε-caprolactone) nanocapsules (CLP and CNLP, respectively), from CARBOPOL hydrogels across porcine buccal and palatal mucosal epithelium, in comparison to EMLA.

Epithelium	Local anesthetic	Formulation	J_ss_ (µg.cm^−2^.h^−1^)	Lag time (h)	R^2^
Buccal mucosa	LDC	CNLP	160.88 ± 31.20^a^	0.57 ± 0.11^a^	0.993 ± 0.005
		CLP	248.03 ± 14.60^b^	0.34 ± 0.20^a.b^	0.997 ± 0.002
		EMLA	280.32 ± 44.43^b^	0.09 ± 0.04^b^	0.997 ± 0.002
	PLC	CNLP	172.24 ± 20.99^c^	0.50 ± 0.15	0.993 ± 0.008
		CLP	168.14 ± 20.40^c^	0.28 ± 0.09	0.997 ± 0.003
		EMLA	283.27 ± 43.46^d^	0.27 ± 0.14	0.995 ± 0.002
Palatal mucosa	LDC	CNLP	119.63 ± 8.83^a^	0.00 ± 0.00	0.991 ± 0.003
		CLP	207.18 ± 25.79^b^	0.21 ± 0.21^a^	0.996 ± 0.003
		EMLA	316.82 ± 16.93^c^	0.00 ± 0.00	0.994 ± 0.003
	PLC	CNLP	92.10 ± 8.75^d^	0.00 ± 0.00	0.994 ± 0.002
		CLP	118.06 ± 13.05^e^	0.17 ± 0.05^#^	0.995 ± 0.006
		EMLA	338.33 ± 16.3 ^f^	0.00 ± 0.00	0.995 ± 0.004

PLC: prilocaine; LDC: lidocaine; J_ss_: steady state flux; R^2^: coefficient of determination of the linear regression model. Different letters indicate a significant difference (*p* < 0.05, ANOVA/Tukey test). Each permeation parameter was analyzed separately for each local anesthetic. Mean ± SD (n = 6).

Due to its similarity with human tissues in terms of structure, lipids, and permeability, porcine buccal mucosa is frequently used for *in vitro* drug permeation studies^49^. The palatal mucosa was selected because of its keratinized layer, which provides a more effective permeation barrier and simulates the application site used in clinical studies^50^.

The fluxes of the two LAs were significantly lower (*p* < 0.05) for CNLP than for the commercial EMLA formulation. This could be explained by the high degree of encapsulation of the drugs in the PCL nanocapsules (Table 2), which decreased the supply of the free drugs crossing the barrier. In addition, the permeation fluxes were influenced by the nature and composition of the formulations tested, which altered the solubility and partitioning of the drugs, compared to the commercial formulation, which is a cream^18,51,52^.

Considering the fluxes of the LAs across the buccal mucosa, LDC showed a significant effect of encapsulation, since both CLP and EMLA presented higher LDC fluxes (*p* > 0.05), compared to CNLP, suggesting that the high encapsulation degree of LDC (83%) reduced its rate of transfer across the barrier, as reported elsewhere^53,54^. On the other hand, the PLC flux was only influenced by the composition of the formulations, as evidenced by the lower fluxes observed for both CNLP and CLP (*p* < 0.05), compared to EMLA.

For the palatal mucosa, both LAs presented fluxes in the decreasing order EMLA > CLP > CNLP (*p* < 0.05) (Table 2), suggesting that for the keratinized barrier model, the composition and nature of the formulation significantly influenced transport of the drugs through the barrier. The higher rate of permeation of EMLA across the keratinized barrier could be explained by the presence of polyoxyl hydrogenated castor oil surfactant in the formulation (EMLA cream, 5 mg/g, AstraZeneca), which was found by Moghadam *et al*.^52^ to disorganize the lipid barrier of the stratum corneum, hence facilitating drug transfer.

The lag times obtained for the LAs with the buccal mucosa (Table 2) showed no difference between LDC in CNLP and in CLP (*p* > 0.05). However, both hydrogels showed slower transfer of LDC, compared to EMLA (*p* < 0.05). On the other hand, the transfer of PLC showed no influence of formulation or encapsulation (*p* > 0.05). Considering the palatal mucosa (Table 2), both LDC and PLC presented a longer lag time when present in the CLP formulation (*p* < 0.01), while their permeation was immediate when present in the CNLP and EMLA formulations. This feature could result in faster onset of topical anesthesia in keratinized mucosa. In previous work, permeation parameters determined *in vitro* were correlated to the efficacy (in humans) of topical anesthetics present in a liposomal CARBOPOL system^18,55^.

#### *In vitro* cytotoxicity assays

**Figure 4:**
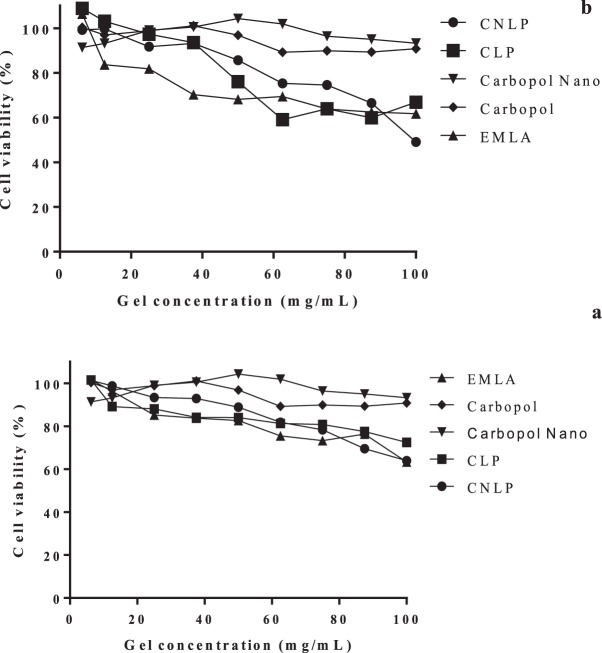
Cell viability determined using the MTT test after exposure of (**a**) FGH and (**b**) HaCaT cells to the hydrogel formulations (n = 9).

Biological studies are essential for provision of detailed information about the contact of drugs with the oral mucosa surface. In viability tests, FGH and HaCaT cells are considered important cellular models for the testing of potential irritants^56^. The results of the cell viability tests are shown in Fig. 4(a,b).

Encapsulation of the LAs in PCL nanocapsules (CNLP) reduced the cytotoxicity towards the HaCaT and FGH cells, compared to the non-encapsulated hydrogel (CLP) or the commercial formulation (*p* > 0.05). In previous work, our research group demonstrated the protective effects of polymeric nanocapsules in hydrogels, for the cytotoxicity of ATC towards 3T3 fibroblasts^5^, and of nanostructured lipid carriers in hydrogels, for the cytotoxicity of LDC-PLC towards 3T3 fibroblasts, HaCaT, and VERO cells^57^. This protective effect was not demonstrated in the present study.

#### Evaluation of *in vivo* anesthetic efficacy

**Figure 5:**
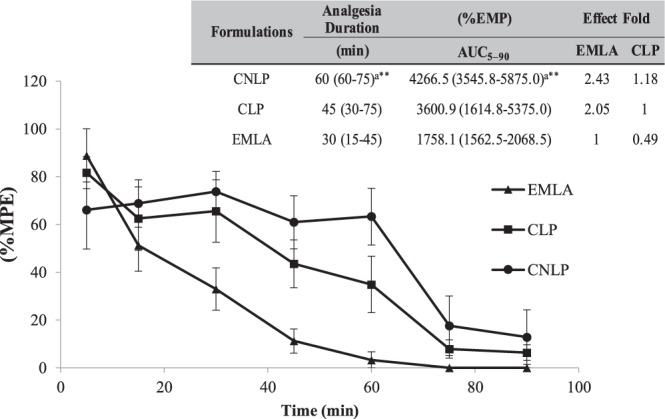
Analgesia time courses, durations, areas under the efficacy curves (AUC_5–90_), and effect ratios for the tail-flick tests employing the different hydrogels. The data are shown as a percentage of the maximum possible effect (% MPE). **p < 0.01^a^ CNLP versus EMLA; Kruskal-Wallis/Dunn test. Median (minimum-maximum) (n = 6). The effect ratios were calculated by dividing the AUC for CNLP or CLP by the AUC for EMLA.

The tail-flick test is an *in vivo* model commonly reported in the literature as being effective for evaluation of the antinociceptive activity of topical formulations^51^, so it was therefore selected here for determination of the effectiveness of the hydrogels. The anesthetic efficacy results are shown in Fig. 5.

The CNLP formulation showed significant improvements in the duration of anesthesia (*p* < 0.01) and the maximum possible effect (*p < *0.01), compared to the commercial formulation (Fig. 5). Considering the AUC_5–90_, CNLP presented a 2.43-fold higher anesthetic efficacy, compared to the commercial formulation. Sharma *et al*.^58^ found that the eutectic LDC-PLC (5%) mixture associated with a thermoresponsive mixed micellar nanogel, used for topical anesthesia, presented a 1.25-fold greater effect, relative to EMLA, supporting the present findings. Puglia *et al*.^54^ reported lower permeation flux and higher anesthetic activity for an LDC formulation associated with nanostructured lipid carriers, compared to the non-encapsulated drug.

Considering that the non-encapsulated formulations showed faster initial action, as observed for the maximum possible effect at the first evaluation time (Fig. 5), the improved anesthetic efficacy of the CNLP hydrogel was probably due to reduced loss of the LAs to the systemic circulation. Consequently, the encapsulation of the drugs acted to increase the residence time of effective amounts of the compounds at the free nerve endings.

## Conclusions

Lidocaine (2.5%) and prilocaine (2.5%) were successfully loaded into poly(ε-caprolactone) nanocapsules, resulting in a hydrogel with desirable characteristics including stability and a satisfactory permeability profile. The new formulation showed improved mechanical, rheological, and mucoadhesive properties, together with lower cytotoxicity and higher *in vivo* anesthetic efficacy, compared to the commercial product. The formulation developed in this work has the potential to provide effective and longer-lasting superficial anesthesia at the intraoral mucosa during medical and dental procedures. These results open perspectives for future clinical trials.

## Materials and Methods

### Chemical agents

Local anesthetics in the base form (LDC and PLC) and capric/caprylic triglyceride (MYRITOL 318) were kindly donated by Cristália (Itapira, SP, Brazil) and Chemspecs (São Paulo, SP, Brazil), respectively. CARBOPOL ULTREZ 10 was purchased from Lubrizol (San Diego, CA, USA); NIPAGIN from Chemco (Hortolândia, SP, Brazil); acetone and chloroform from Synth (Labsynth, Diadema, SP, Brazil); poly(vinyl alcohol) (PVA, MW 30,000-70,000), polycaprolactone (PCL, MW 70,000-90,000), triethanolamine, glycerine, phosphoric acid, ammonium hydroxide, uranyl acetate, polysorbate 80 (Tween 80), and MTT Assays from Sigma-Aldrich (Merck KGaA, Darmstadt, Germany); acetonitrile from J.T. Baker (Phillipsburg, NJ, USA); anhydrous dibasic sodium phosphate, anhydrous monobasic sodium phosphate, anhydrous monobasic potassium phosphate, sodium chloride, and potassium chloride from Dinâmica (Dinâmica Química Contemporânea, Diadema, SP, Brazil). Ultrapure water from a Milli-Q system (Millipore, Bedford, MA, USA) was used to prepare all solutions. The commercially available topical anesthetic product composed of the eutectic mixture of LDC and PLC (5%) (EMLA cream, AstraZeneca, Brazil) was used as the positive control in some experiments, as detailed below.

### Analytical procedures

Simultaneous quantification of LDC and PLC was performed using an HPLC system (Thermo Electron Corporation, Waltham, MA, USA) equipped with an automatic sampler (Surveyor Autosampler Plus Lite, Thermo Electron Corporation) and a Surveyor UV-VIS Plus detector (Thermo Electron Corporation). ChromeQuest 5.0 software (Thermo Fisher Scientific, Waltham, MA, USA) was used for data collection. The local anesthetics were separated on a Phenomenex Gemini C18 reversed-phase column (5 µm, 150 × 4.60 mm). The mobile phase consisted of a mixture of acetonitrile:buffer (25 mM NH_4_OH, pH 7.0, adjusted with H_3_PO_4_) at a ratio of 40:60 (v:v), pumped at 1.2 mL/min. The injection volume was 200 µL. Detection of the LAs was performed at a wavelength of 220 nm^18^.

### Preparation of poly(ε-caprolactone) (PCL) nanocapsules containing 2.5% lidocaine (LDC) and 2.5% prilocaine (PLC)

Polymeric nanocapsules composed of poly(ε-caprolactone) (PCL) and containing the eutectic mixture of LDC (2.5%) and PLC (2.5%) (PCL/LDC + PLC) were prepared using an oil-in-water emulsion/solvent evaporation method^11^. Briefly, LDC (250 mg), PLC (250 mg), and capric/caprylic triglycerides (200 mg) were dissolved in acetone (10 mL) and mixed with chloroform (20 mL) containing the polymer (400 mg), followed by ultrasonication (1 min, 90 W). The aqueous phase (50 mL) containing PVA (175 mg) was then added to the pre-emulsion obtained and the mixture was ultrasonicated (8 min, 90 W). The resulting emulsion was rotary-evaporated to 5 mL, in order to remove the solvents. The resultant PCL/LDC + PLC samples were stored in a dryer at 4 °C before use.

### Preparation of porcine oral mucosa

Porcine oral mucosa was prepared according to the methodology described previously^50^. Pig maxillae (from 5-months-old Landrace pigs weighing around 75–80 kg) were obtained in a local slaughterhouse, immediately after slaughter, stored in ice-cold isotonic phosphate buffer (pH 7.4), and transported to the laboratory within 1 h.

For the *in vitro* permeation studies, samples of palatal (keratinized mucosa model) and buccal (from the cheek region, non-keratinized mucosa model) mucosa were separated from the underlying tissue using a scalpel and were washed with saline. Intact mucosa was immersed in deionized water at 60 °C for 2 min. The epithelium was carefully separated from the connective tissue and was used immediately^50^.

In the mucoadhesive strength experiment, porcine buccal mucosa from the cheek was used. The cheek was removed with a scalpel and washed in distilled water. The underlying muscle layer was smoothed and retained, in order to support the mucosa. The tissues were kept in isotonic phosphate buffer (pH 7.4) and were rapidly used in the experiments^40^.

All the experiments involving porcine oral mucosa were performed with mucosa from at least three different animals.

### Characterization of poly(ε-caprolactone) nanocapsules

#### Determination of particle size, polydispersity, and surface charge

The nanoparticle average diameter (nm) and polydispersity index (PDI), determined by dynamic light scattering (DLS), together with the surface charge (zeta potential, ZP), were measured using a Zetasizer ZS-90 particle analyzer (Malvern Instruments, Malvern, UK). The PCL suspensions (with and without LDC + PLC) were analyzed in triplicate at 25 °C, on three different days, using a scattering angle of 90°^11^.

#### Nanoparticle concentration

The nanoparticle concentration was determined by nanoparticle tracking analysis (NTA), using an LM20 instrument (NanoSight, Amesbury, UK) equipped with a 532 nm laser. The nanoparticle concentration (particles/mL) was obtained in real time, at room temperature, based on light scattering and the individual Brownian motion tracks of the nanoparticles^28^. Using a sterile syringe, the formulation was injected into the sample chamber until the liquid filled the tip of the syringe. The measurements (n = 3) were performed over a period of 6 months.

#### Physicochemical stability of nanoparticles in suspension

The stabilities of the formulations containing PLC and LDC were evaluated by measuring the nanoparticle size, PDI, ZP, concentration, and pH after 7, 15, 30, 60, 90, 120, 150, and 180 days of storage at room temperature (25 °C)^59^.

#### Structural analyses

Attenuated total reflectance-Fourier transform infrared (ATR-FTIR) analysis: Infrared analyses (ATR-FTIR) were performed of the PCL nanocapsules with and without LDC + PLC. LDC and PLC were also analyzed in their free forms. The spectra were obtained using FTIR spectrophotometers (Bruker IFS 66 v/S or Perkin Elmer Spectrum 65) fitted with ATR cells and operated in reflectance mode, in the range 4500–500 cm^−1^, with steps of 2 cm^−1^.

Differential scanning calorimetry (DSC) analysis: The same formulations were also submitted to DSC measurements performed using a TA Q20 calorimeter equipped with a cooling system. After calibrating the equipment with indium, 5 mg portions of the samples were placed in aluminum pans and the thermal profiles were obtained in the temperature range from 0 to 250 °C, at a heating rate of 10 °C/min, under a flow of nitrogen.

#### Determination of nanoparticle morphology

The morphologies of the PCL nanoparticles with and without LDC + PLC were evaluated by transmission electron microscopy (TEM), using an EM-900 instrument (Carl Zeiss, Jena, Germany) operated at 80 kV. Briefly, the samples were diluted, deposited onto copper grids coated with carbon film, contrasted using uranyl acetate (2%), dried at room temperature, and analyzed.

#### Encapsulation efficiency (EE%)

The encapsulation efficiencies of LDC and PLC in PCL were determined by the method combining ultrafiltration and centrifugation^11^. The EE% values were determined from the difference between the total and free LDC and PLC concentrations. The PCL/LDC + PLC samples were centrifuged for 15 min, at 28000 *g*, in regenerated cellulose Microcon ultrafiltration units with a molecular exclusion pore size of 30 kDa (Millipore, Billerica, Massachusetts), followed by quantification of the compounds in the suspension using HPLC.

#### *In vitro* release kinetics

The profiles of *in vitro* release of PLC and LDC from the PCL nanoparticles were investigated using a two-compartment system consisting of donor (1 mL) and acceptor (80 mL) compartments, separated by a cellulose membrane with a molecular exclusion pore size of 10 kDa^11^. The system was maintained under sink conditions, with constant magnetic stirring (300 rpm) at room temperature (25 °C). Aliquots of 300 μL were periodically withdrawn from the acceptor compartment, over a total period of 1400 min. The samples were analyzed by HPLC. The experiment was performed in triplicate. The data were treated by the application of different mathematical models in order to select the best model (defined by the highest R^2^ value) to describe the LA release mechanism. The software used was KinetDS 3.0 (Jagiellonian University Medical College, Kraków, Poland).

### Hydrogel preparation

The CARBOPOL (carboxyvinyl derivative) hydrogel base (**C**) was prepared according to the technique described previously^60^, using the following components: CARBOPOL (2%, gelling agent); propylene glycol (5%, solvent and wetting); methylparaben (0.2%, preservative); glycerin (8%, wetting and emollient agent); deionized water (solvent); triethanolamine (pH 7,0, alkalinizing agent). For preparation of the CARBOPOL hydrogel with or without 2.5% LDC and 2.5% PLC associated with the poly(ε-caprolactone) nanocapsules, the resulting hydrogel (placebo) was immediately mixed with nanocapsule suspensions with (**CNLP**) or without (**CN**) the local anesthetics (50:50, v/v) at the desired final drug concentration (5% w/w LDC + PLC). CARBOPOL hydrogel containing free 2.5% LDC and 2.5% PLC (**CLP**) was prepared by dissolving appropriate amounts of the local anesthetics (5% w/w LDC + PLC) in the propylene glycol during hydrogel preparation.

### Characterization of the hydrogel formulations

#### Rheological measurements

Rheological measurements were performed using a Haake RheoStress 1 rheometer (Thermo-Haake, Germany) with plate-plate geometry (plate diameter of 20 mm). The hydrogel formulations were submitted to continuous variation of shear rate from 0 to 300 s^−1^, and the resulting shear stress was measured. The tests were performed in triplicate (n = 3) at a constant temperature (25 ± 1 °C) maintained by a thermostatically-controlled water bath. The rheological behaviors of the hydrogel formulations were evaluated from curves obtained by plotting the shear stress (Pa) as a function of shear rate (s^−1^)^11^.

#### Mechanical properties of the hydrogels

The mechanical properties (hardness, compressibility, cohesiveness, and adhesiveness) of the hydrogel formulations were determined using a texture analyzer (Model TA-XT Plus, Stable Micro Systems) operated in texture profile analysis (TPA) mode. A 10 mL beaker was filled with approximately 10 g of each formulation (to a fixed height) and the samples were left in a water bath at 37 °C for 24 h for removal of air bubbles. The analytical probe (10 mm diameter) was compressed twice into each sample, to a depth of 5 mm, at a rate of 2.0 mm.s^−1^, with a delay period of 15 s between the end of the first compression and the beginning of the second compression.

#### Accelerated stability study

The hydrogels were placed in a stability chamber with controlled temperature and humidity (40 °C and 75% relative humidity). The parameters evaluated were the anesthetic dosage, pH, and weight loss^46^.

Determination of the dosages of lidocaine and prilocaine in the formulations: The dosages of the LAs in the hydrogels were measured by diluting appropriate amounts of the semisolid formulations, followed by HPLC analyses, as described previously.

pH analysis: The pH values of the hydrogel formulations were determined by potentiometric measurements using an Analyzer Model 300 M pH meter equipped with an electrode for semisolids.

Weight loss test: The percentage weight loss throughout the study was calculated using the following equation:

$$ \% \,{\rm{weight}}\,{\rm{loss}}=[({\rm{MWI}}-{\rm{MWS}})\times 3]/{\rm{MWI}}\times {\rm{100}}$$

where MWI is the mean initial weight, MWS is the mean weight at subsequent times (3 or 6 months), and 3 is the correction factor to 75% RH^46^.

#### *In vitro* release kinetics of the hydrogels and mathematical modeling

The release of the LAs from the hydrogels (CLP and CNLP) was evaluated using a Franz-type vertical diffusion system (Manual Transdermal System, Hanson Research Corporation, Chatsworth, CA, USA) with permeation area of 1.77 cm^2^ and a 7 mL volume acceptor chamber. The hydrogels (CNLP and CLP) were applied under infinite dose conditions (using 300 mg) over a dialysis membrane with a molecular exclusion pore size of 1,000 Da. The system was maintained at 37 °C, under magnetic stirring (300 rpm)^11^. Aliquots of 300 µL were withdrawn from the acceptor compartment for HPLC analysis, with the volume being maintained constant by addition of the same volume of fresh buffer solution. Samples were collected at predetermined intervals during a period of 24 h, in triplicate.

KinetDS 3.0 software was used to analyze the release curves and the best kinetic model was selected based on the coefficient of determination (R^2^).

#### *In vitro* permeation studies

*In vitro* permeation assays were performed with porcine buccal and palatal mucosal epithelium, using the same Franz-type vertical diffusion system described above^50^.

The epithelium was positioned on a 0.45 μm cellulose filter with the connective side of the tissue facing the filter, due to its fragility, hence reducing the release of impurities into the acceptor compartment, without altering the permeation of the LAs^50^.

The hydrogels (CNLP and CLP), epithelium, and membrane filter were clamped between the donor and acceptor compartments. The acceptor compartment was filled with PBS:alcohol (70:30, v/v) that had been filtered and degassed. The acceptor medium was selected according to the solubility of the LAs, in order to maintain sink conditions whereby the concentrations of the drugs in this compartment never reached 10% of their solubility (LDC = 17.95 mg/mL; PLC = 21.89 mg/mL).

The experiment was performed at 37 °C during 5 h, under magnetic stirring (350 rpm). At predetermined intervals, 300 μL volumes of the samples were collected and the same volumes of fresh acceptor medium were added. The samples were analyzed by HPLC, as described previously.

For each cell, the cumulative amounts of the LAs transported across the mucosal epithelium, per unit of area, was plotted against time. The steady-state fluxes (J_ss_) of the LAs were calculated from the slopes of the linear portions of the curves. The lag times were obtained from the intercepts on the time axis. All experiments were conducted six times^50^.

### *In vitro* evaluation of mucoadhesive strength

The mucoadhesive strengths of the formulations were evaluated by measuring the force required to detach them from pig buccal mucosa, using the same texture analyzer described above, in accordance with Cubayachi *et al*.^40^. The buccal mucosa was positioned horizontally at the lower end of the TPA probe and the formulation was placed at the upper end. Prior to the mucoadhesion testing, the buccal mucosa was hydrated with 50 µL of artificial saliva for 5 min. The analytical probe was then lowered until it made contact with the mucosa surface. The rupture tensile strength was determined by applying a compressive force of 0.5 N for 30 s and then moving the probe at a constant speed of 1.0 mm.s^−1^. The force required to detach the formulation from the mucosa surface was determined from the curve of force plotted against distance. All the measurements were performed at ambient temperature (21 ± 1 °C), in quintuplicate.

### *In vitro* cytotoxicity assays

The cytotoxicities of the hydrogel formulations were evaluated by the MTT (a yellow tetrazole) reduction test with human epithelial cells (HaCaT) and human gingival fibroblasts (FGH). Viable cells were exposed for 2 h to the hydrogel formulations (CNLP and CLP) containing the local anesthetics at different concentrations (ranging from 0.312 to 5 mg/mL of LDC and PLC). The formulations contained from 6.25 to 100 mg/mL of hydrogel. The same amounts of hydrogel (6.25 to 100 mg/mL) free of local anesthetics (CN and C) were diluted and used as placebos. The percentages of viable cells were determined after incubation in the presence of MTT for 3 h at 37 °C, with measurement of the amount of MTT converted to insoluble formazan by mitochondrial dehydrogenases^61,62^. A 100 μL volume of ethanol was added to each well in order to dissolve the formazan crystals, resulting in a purple solution.

The fraction of viable cells was obtained by quantification of the original formazan using a microplate reader (BioTek Instruments Inc., Winooski, VT, USA) operated at a wavelength of 570 nm, with conversion of the value to the percentage of viable cells.

### *In vivo* anesthetic efficacy evaluation

The experiments were conducted after approval by the Animal Ethics Committee of UNICAMP (protocol #2850-1) and in accordance with the Principles of Laboratory Animal Care (NIH publication #85-23, revised in 1985). Male adult Wistar rats (200–250 g) obtained from the Multidisciplinary Center of Biological Investigation of Laboratory Animals (CEMIB-UNICAMP) were kept in cages under light/dark cycles of 12 h, at 25 ± 2 °C, and were provided with water and food ad libitum for at least 7 days before the experiments. The Wistar rats were divided into groups of 6 animals and each animal was used only once in the experiment.

The topical anesthetic efficacies of the hydrogels containing LDC and PLC were assessed using the tail-flick test, as previously described by Grant *et al*.^63^ and modified by De Araujo *et al*.^51^. Briefly, the animals were positioned in an acrylic confinement chamber, while maintaining free the distal portion of the tail (5 cm). The time required for tail removal (latency) following exposure to the heat produced by an incandescent lamp (55 °C) was considered as the aversive response, generating a baseline that was recorded for each animal prior to the start of the experiment. In order to avoid injury due to thermal insult, a maximum time of 15 s (cut-off value) was established for contact with the heat source. Approximately 0.5 g portions of the hydrogels and the commercial formulation (EMLA) were applied 2 cm from the tail base, with the aid of MICROPORE protective tape, for 2 min. The formulations were then removed and nociceptive stimulus was applied to the same region. Measurements were performed immediately after formulation removal and then every 15 min until the animal returned to its baseline pain response. After the tail-flick test, the animals were sacrificed by deep general anesthesia. Analgesia was defined as an at least 50% increase in the time required for tail removal, compared to the observed baseline value. Calculation of the maximum possible effect (%MPE) was performed by subtracting the baseline from the latency time and dividing the resulting value by the cut-off minus the baseline. The value obtained was multiplied by 100.

### Data analysis

The results from the suspension and hydrogel characterizations, the *in vitro* permeation tests, and the mucoadhesion tests were statistically evaluated using the Student’s t-test, or by analysis of variance (ANOVA) followed by Tukey’s post-hoc test. These analyses were performed with BIOSTAT 5.0 for Windows software (Instituto Mamirauá, Belém, PA, Brazil). The results of the *in vitro* cytotoxicity assays and the *in vivo* tests were analyzed using the Kruskal-Wallis test followed by the Dunn post-hoc test. These analyses were performed with GraphPad Prism 6.0 software (GraphPad, San Diego, CA). Statistical significance was defined as *p* < 0.05.

## Electronic supplementary material

Supplementary information

## Data Availability

All data generated or analyzed during this study are included in this manuscript.
