# Supplementary material for: Hybrid Hydrogel Composed of Polymeric Nanocapsules Co-Loading Lidocaine and Prilocaine for Topical Intraoral Anesthesia
Source: Sci Rep. 2018 Dec 19;8:17972. doi: 10.1038/s41598-018-36382-4 (PMC6299281; doi:10.1038/s41598-018-36382-4)
Supplement: Supplementary file 1 — Supplementary information [file 41598_2018_36382_MOESM1_ESM.docx]

# Supplementary Information

# Hybrid Hydrogel Composed of Polymeric Nanocapsules Co-Loading Lidocaine and Prilocaine For Topical Intraoral Anesthesia

**Bruno Vilela Muniz¹, Diego Baratelli², Stephany Di Carla¹, Luciano Serpe¹, Camila Batista da Silva¹, Viviane Aparecida Guilherme^3^, Lígia Nunes de Morais Ribeiro^3^, Cintia Maria Saia Cereda^3^, Eneida de Paula³, Maria Cristina Volpato^1^, Francisco Carlos Groppo¹, Leonardo Fernandes Fraceto², Michelle Franz-Montan^1^***

^1^ Department of Physiological Sciences, Piracicaba Dental School, University of Campinas – UNICAMP, Piracicaba, São Paulo, Brazil

² São Paulo State University – UNESP, Institute of Science and Technology of Sorocaba, Department of Environmental Engineering, Sorocaba, São Paulo, Brazil

³ Department of Biochemistry and Tissue Biology, Institute of Biology, University of Campinas – UNICAMP, Campinas, São Paulo, Brazil

*Corresponding author:

Michelle Franz-Montan

Av. Limeira 901, Bairro Areião, 13414-903, Piracicaba, São Paulo, Brazil

Tel.: 55 19 2106 5306; FAX: 55 19 2106 5306; E-mail: [michelle@fop.unicamp.br](mailto:michelle@fop.unicamp.br)


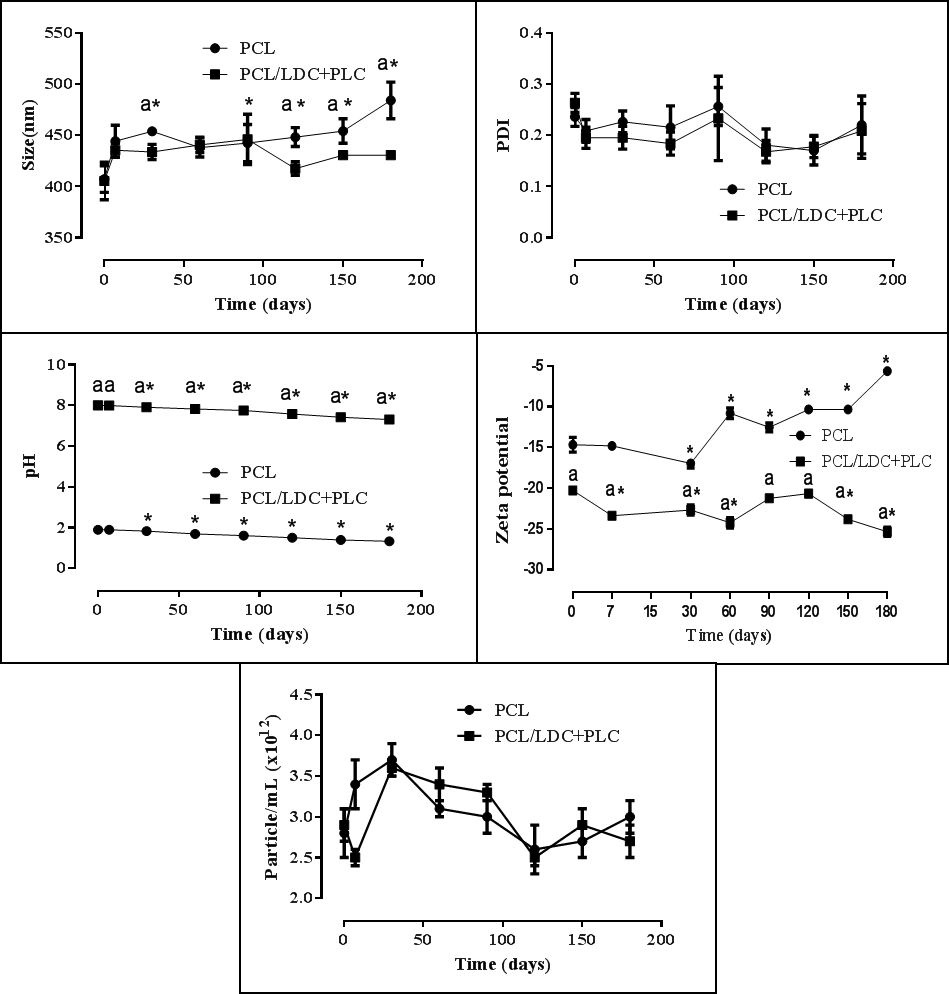


**Figure S1.** Mean (±SD) size (nm), PDI, pH, surface charge (ZP), and nanoparticle concentration (.10^12^ part./mL) of poly(ε-caprolactone) nanocapsules (PCL) and nanocapsules containing lidocaine-prilocaine (PCL/LDC+PLC), according to time, during 180 days of storage at 25 ^o^C. Comparison between suspensions with or without LAs (PCL/LDC+PLC *vs.* PCL) for each period: Student’s t-test (*p* < 0.01). Variation of formulation parameters over time (relative to Day 0): ANOVA/Tukey’s test (*p* < 0.05; n = 3).


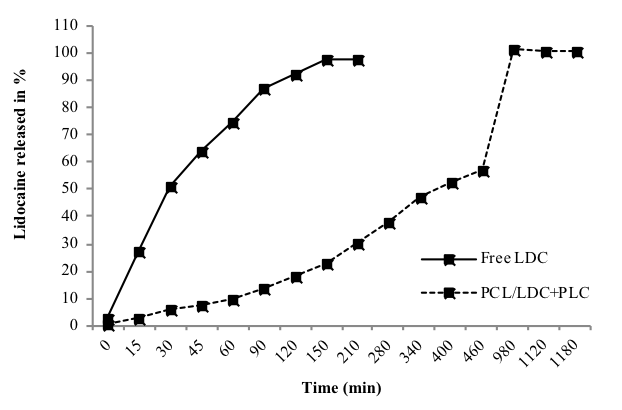

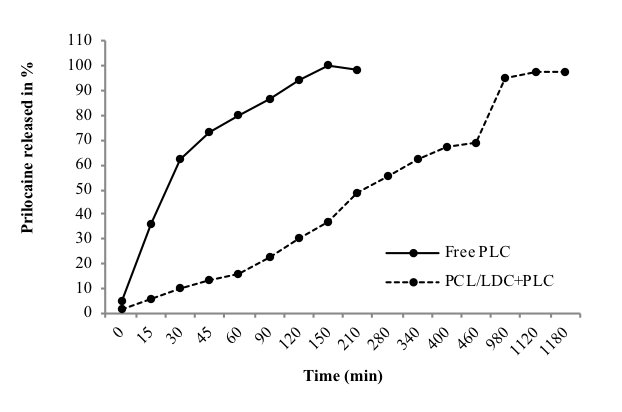


**b**

**a**

**Figure S2.** Percentages of (a) lidocaine (LDC) and (b) prilocaine (PLC) released from free solution and poly(ε-caprolactone) nanocapsules (PCL/LDC+PLC), at 25 ºC (n = 3). SD values were below 5%.

**Figure S3**. Mean (±SD) percentages of (a) lidocaine and (b) prilocaine released from Carbopol^®^ hydrogels containing LDC and PLC (5%), free or associated with poly(ε-caprolactone) nanocapsules (CLP and CNLP, respectively), according to time, at 37 ºC (n = 3).


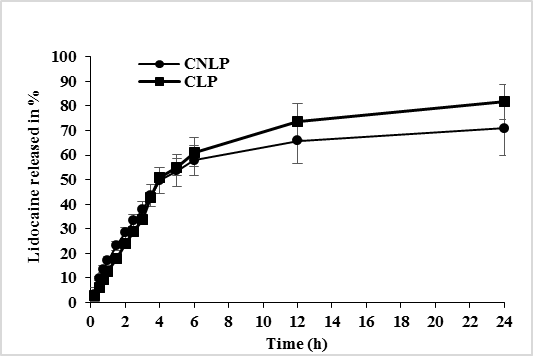

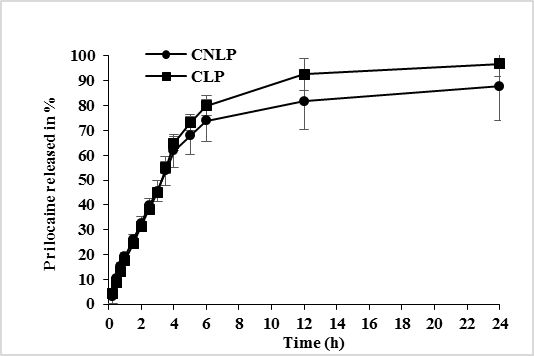


**a**

**b**

**Table S1.** Correlation coefficient values obtained for lidocaine (LDC) and prilocaine (PLC), using different mathematical models applied to analyze the kinetics of release from the nanocapsules.

| **Release kinetics** | | Korsmeyer-Peppas | | | Weibull | Zero order |
| --- | --- | --- | --- | --- | --- | --- |
|  | | R^2^ | k | n | R^2^ | R^2^ |
| **Lidocaine** | PCL/LDC+PLC | 0.99 | 0.003 | 0.82 | 0.89 | 0.70 |
| **Prilocaine** | PCL/LDC+PLC | 0.98 | 0.007 | 0.72 | 0.88 | 0.66 |

**Table S2.** Mean (±SD) local anesthetic content (dosage), pH, and weight variation of the different Carbopol^®^ hydrogels containing LDC and PLC (5%), free or associated with poly(ε-caprolactone) nanocapsules (CLP and CNLP, respectively), compared to EMLA^®^, during 6 months storage at 40 ± 2 °C and 75% RH.

| **Hydrogel formulation** | **Time**  **(months)** | **Dosage** | | **pH** | **ΔP** |
| --- | --- | --- | --- | --- | --- |
|  |  | **LDC** | **PLC** |  |  |
| CNLP | T0 | 2.77 ± 0.09 | 2.02 ± 0.04 | 7.324 ± 0.24 | ------ |
|  | T3 | 1.91 ± 0.03^a^ | 1.88 ± 0.03 | 7.100 ± 0.26 | 0.11 ± 0.03 |
|  | T6 | 2.44 ± 0.37 | 1.87 ± 0.30 | 7.310 ± 0.09 | 0.14 ± 0.04 |
| CLP | T0 | 2.77 ± 0.07 | 2.50 ± 0.09 | 7.482 ± 0.07 | ------ |
|  | T3 | 2.34 ± 0.02ª | 2.17 ± 0.05^a^ | 4.831 ± 0.11ª | 0.09 ± 0.01 |
|  | T6 | 2.69 ± 0.02^b^ | 2.20 ± 0.09ª | 8.405± 0.21^a,b^ | 0.01 ± 0.01* |
| EMLA^®^ | T0 | 3.15 ± 0.01 | 2.49 ±0.02 | 8.945 ± 0.26 | ------ |
|  | T3 | 2.29 ± 0.05^a^ | 2.30 ± 0.04 | 9.024 ± 0.29 | 0.27 ± 0.44 |
|  | T6 | 2.84 ± 0.35^b^ | 2.5 ± 0.30 | 9.423 ± 0.26 | 0.01 ± 0.03 |

T0: time zero; T3: 3 months; T6: 6 months; ΔP: weight variation for T3 and T6, relative to T0 and T3, respectively; **p* < 0.01. Different letters indicate statistically significant differences among the times (*p* < 0.05): ^a^ relative to T0; ^b^ relative to T3 (ANOVA/Tukey’s test; n = 3).

**Table S3.** Release kinetics mechanisms for lidocaine and prilocaine, free or associated with poly(ε-caprolactone) nanocapsules (CLP and CNLP, respectively), contained in Carbopol^®^ hydrogels, as evaluated by the determination coefficient values (R^2^) obtained following application of different mathematical models.

|  | Release kinetics | Weibull | | | Korsmeyer-Peppas | Zero order |
| --- | --- | --- | --- | --- | --- | --- |
|  |  | R^2^ | K | b | R^2^ | R^2^ |
| **Lidocaine** | CNLP | 0.96 | 0.49 | 0.72 | 0.89 | 0.61 |
|  | CLP | 0.95 | 0.72 | 0.95 | 0.93 | 0.68 |
| **Prilocaine** | CNLP | 0.94 | 0.50 | 0.93 | 0.72 | 0.60 |
|  | CLP | 0.94 | 0.54 | 0.13 | 0.74 | 0.65 |
